# Editorial: Genetics of female infertility

**DOI:** 10.3389/fgene.2023.1297173

**Published:** 2023-09-29

**Authors:** Masood Bazrgar, Hamid Gourabi

**Affiliations:** Department of Genetics, Reproductive Biomedicine Research Center, Royan Institute for Reproductive Biomedicine, Tehran, Iran

**Keywords:** female infertility, genetics, endometriosis, PCOS, POI, fertilization failure, recurrent miscarriage

The prevalence of reproductive disorders and infertility affects approximately 10%–15% of couples worldwide, with a similar contribution from both genders. Female infertility (FI) has a complex multifactorial origin, involving numerous genes associated with sex determination, gametogenesis, fertilization, hormonal interactions, embryo implantation, and early development. Dysfunctions or failures at any stage of these processes can be linked to genetic factors. In addition to DNA sequence variations, non-coding RNAs and epigenetic modifications may also be associated with FI. Chromosomal abnormalities are known causes of conditions such as premature ovarian insufficiency (POI), recurrent miscarriages, and implantation failure. POI is clinically heterogeneous and often associated with ovarian dysgenesis. Some progress has been made in understanding the genetic basis of common multifactorial disorders like polycystic ovary syndrome (PCOS) and endometriosis, which each affect approximately 10% of women with infertility or subfertility. Pharmacogenomics research has also advanced, aiming to identify genetic variations that influence individual responses to controlled ovarian stimulation, aligning with the principles of personalized medicine. However, in conditions like endometriosis, PCOS, and POI, which are the most common causes of FI, there are still many unrevealed genetic contributions. The aim of this Research Topic is to provide novel evidence and review existing data on the genetics of FI, leading to a comprehensive understanding of its etiology and pathogenesis. This knowledge will facilitate the development of targeted diagnostic and therapeutic strategies. Additionally, we aim to identify gaps in the current research and studies in this field that have not yet been addressed.

This Research Topic comprises seven articles, including four original research papers, two case reports, and a systematic review with meta-analysis.

Huo et al. investigated the gene spectrum in nine patients with oocyte maturation arrest. They identified nine pathogenic variants in five genes, including three novel variants: *PATL2* [c.1374A > G (p. Ile458Met)] and [1289-1291del TCC (p. Leu430del)], and *ZP2* [c.1543C > T (p. Pro515Ser)]. The identified pathogenic mutations in *PATL2*, *TUBB8*, and *ZP1*∼3 genes strongly suggest their involvement in oocyte maturation arrest. In another study, Zhou et al. predicted pathogenic mutation sites in the *PATL2* gene and estimated the carrier frequency of these mutations to be at least 1.14% based on the gnomAD and ExAC databases. Phenotypic variability was found to be associated with the functional regions and the degree of loss of function of the PATL2 protein. The estimated allele and carrier frequencies were higher than those previously predicted based on clinical ascertainment. The authors reanalyzed data on pathogenic *PATL2* mutant lineages in 34 patients identified by other authors and reported that 53.81% of the oocytes with *PATL2* mutations were arrested at the germinal vesicle (GV) stage, 9.22% at the metaphase I (MI) stage, and 14.72% at the first polar body stage. In a case report, Zhang et al. identified a complex heterozygous mutation in the *PADI6* gene (c.1247T>C; c.2009_2010del), which caused arrest in embryos resulting from 13 MII oocytes at the 1- or 2-cell stage.

Diminished ovarian reserve (DOR) is one of the significant causes of FI, which can ultimately lead to POI. Li et al. conducted whole exome sequencing to explore pathogenic variants associated with DOR in twenty young women under 35 years old who were affected by DOR without definite factors damaging ovarian reserve. They identified a set of mutated genes that may be related to DOR, with particular interest in a missense variant in the *GPR84* gene, which was further studied. It was found that this variant promoted the expression of proinflammatory cytokines (TNF-α, IL12B, IL-1β) and chemokines (CCL2, CCL5), as well as the activation of the NF-κB signaling pathway, suggesting a mechanism for non-age-related pathological DOR.

Li et al. conducted a systematic review with meta-analysis to investigate the relationship between Calpain10 (*CAPN10*) polymorphisms and susceptibility to PCOS. *CAPN10* is the first identified susceptibility gene for type 2 diabetes mellitus and is closely related to insulin sensitivity. Insulin resistance plays a crucial role in the pathogenesis of PCOS. The authors analyzed the genotype and allele frequencies of two *CAPN10* polymorphisms (SNP-43 and SNP-44) in a cohort of PCOS patients and healthy controls. They found a significant association between the *CAPN10* SNP-43 polymorphism and PCOS susceptibility, suggesting that this genetic variant may contribute to the development of PCOS by affecting insulin sensitivity.

Some genetic etiologies are shared in both sexes. Jiang et al. reported a novel variant of *DNAAF4* in Primary ciliary dyskinesia (PCD). *DNAAF4* has been previously reported in male and female infertility. PCD is a rare autosomal recessive disorder that affects the structure and function of motile cilia, leading to classic clinical phenotypes, such as situs inversus, chronic sinusitis, bronchiectasis, repeated pneumonia and infertility. They explored the effect of the mutation on DNAAF4 protein from three aspects: protein expression, stability and interaction with downstream DNAAF2 protein through a series of experiments, such as transfection of plasmids and Co-immunoprecipitation. They confirmed that the mutation of *DNAAF4* led to PCD by reducing the stability of DNAAF4 protein, but the expression and function of DNAAF4 protein had not been affected.

In conclusion, although some genes like PATL2 and CAPN10 could be highlighted in FI, a wide variety of genes play a role in each phenotype or in a specific subphenotype of FI. Some genes might contribute to both male and FI. More investigations are still needed to find major genetic contributors in FI. This Research Topic highlights the importance of genetics in understanding the etiology and pathogenesis of FI. The studies included in this Research Topic provide novel insights into the genetic factors implicated in various conditions associated with FI, such as oocyte maturation arrest, diminished ovarian reserve, PCOS, and endometriosis. The identification of specific genetic variants associated with these conditions can enhance our understanding of the underlying mechanisms and facilitate the development of targeted diagnostic and therapeutic approaches. However, further research is still needed to unravel the complex genetic architecture of FI and to identify additional susceptibility genes and pathways involved. Continued efforts in this field will contribute to improved clinical management and personalized treatment options for women affected by infertility.

